# Dysfunction of Inflammatory Pathways and Their Relationship with Anti-Hypothalamic Autoantibodies in Patients with Anorexia Nervosa

**DOI:** 10.3390/nu15092199

**Published:** 2023-05-05

**Authors:** Andrea Amerio, Andrea Escelsior, Eleonora Martino, Antonella Strangio, Costanza Giacomini, Elisa Montagna, Andrea Aguglia, Marina Bellomo, Samir Giuseppe Sukkar, Daniele Saverino

**Affiliations:** 1 Department of Neuroscience, Rehabilitation, Ophthalmology, Genetics, Maternal and Child Health (DINOGMI), Section of Psychiatry, University of Genoa, 16126 Genoa, Italy; 2 IRCCS Ospedale Policlinico San Martino, 16132 Genoa, Italy; 3 Dietetics and Clinical Nutrition Unit, Genoa University, 16132 Genoa, Italy; 4 Department of Experimental Medicine (DiMeS), Section of Human Anatomy, University of Genoa, 16126 Genoa, Italy; 5 Department of Mental Health and Pathological Addictions, Genoa Local Health Authority ASL4, 16043 Chiavari, Italy

**Keywords:** anorexia nervosa, pro-inflammatory cytokines, IL-21, duration of illness

## Abstract

Background: Despite several attempts, the etiopathogenesis of anorexia nervosa (AN) is still unknown. However, the activation of the immune response in neuropsychiatric diseases, including AN, is increasingly evident. We aimed to explore immune response parameters in patients with AN and identify the link between the presence of specific autoantibodies for hypothalamic antigens and the inflammatory response. The relationship between inflammatory markers and the duration of the disease has been also investigated. Methods: Twenty-two patients with AN were included, and none were under psychopharmacological treatment or suffering from autoimmune conditions. Serum concentrations of interleukin (IL)-6, IL-1β, tumor necrosis factor (TNF)-α, transforming growth factor (TGF)-β, and IL-21 were determined by ELISA kits. In addition, autoantibodies against hypothalamic antigens are quantitatively evaluated. Results: IL-6, IL-1 β, TNF-α, and TGF-β are significantly increased in patients with AN. A positive correlation with body mass index and with the amount of autoantibody specific for hypothalamic antigens exists. Notably, a progressive reduction of cytokines correlates with the progression of AN. In addition, IL-21 is increased in the blood of patients with AN and negatively correlates with autoantibody concentrations. Conclusions: This study shows that the increased pro-inflammatory phenotype in patients affected by AN correlates with the concentration of autoantibody specific for hypothalamic antigens. Of interest, the pro-inflammatory state seems to be reduced with duration of AN. In addition, IL-21 could work as a stimulant of the immune response, thus possibly increasing the autoreactivity.

## 1. Introduction

Anorexia nervosa (AN) is a major psychiatric disorder characterised by an intense fear of weight gain associated with an altered body perception. AN induces patients to reduce food intake and, sometimes, to adopt weight-control behaviours, such as excessive exercise or self-induced vomiting. It is often associated with other psychiatric comorbidities, such as mood or anxiety disorders, and it could especially determine medical complications, which compromise the general health of the patient in the most severe forms. AN has a chronic course with frequent relapses, related to high level of disabilities and mortality, especially when not adequately treated [1], due to not only to the medical consequences of malnutrition, cardiological complications, and severe infectious states, but also to suicidal behaviours [2]. 

Individuals diagnosed with a major psychiatric disorder show increased concentration of inflammatory markers, both in the central and peripheral nervous system. However, the relationship between these alterations and the expression of the clinical features of the psychiatric illnesses remain obscure. An example, supporting the idea that an association exists between inflammation and depression, is represented by symptoms caused by interferon (IFN)-α: up to 50% of patients treated with INF-α develop a major depressive episode during the treatment, and these patients often respond to antidepressants [3]. Moreover, being exposed to inflammation during the postnatal period or during first years of childhood has been associated with alterations of cerebral functioning that sometimes are so severe as to determine major psychiatric disorders, such as schizophrenia or autism spectrum disorders [4].

Alterations of neuroendocrine system, dysregulation of neurotransmitter production, and the structural modification of the neuronal network are only a few of the potential mechanisms involved. Furthermore, intestinal microbiota seems to play a role in inflammation and thus in determining alterations in psychopathology [5]. More specifically, it is demonstrated that microbiota can work when several varieties of bacteria coexist in it [6].

When this diversity is threatened, the permeability of the barrier could be compromised, and lesions called “leaky gut” could appear. These lesions represent a gateway for bacteria to enter in the bloodstream and to start an inflammatory process [7]. Even if there are conflicting data, the hypothesis that a reduction in bacteria diversity could lead to altered perception of body image and thus to anorexia and other comorbidities, such as depression, will deepen [8]. The peripheral inflammation is mediated by the so-called “toll like receptors” (TLRs), which are expressed in their active form on the cellular surface, or within the host cells on the organelle biomembranes, such as the endoplasmic reticulum, endosomes, and lysosomes. The bond between TLRs and bacterial products starts the inflammatory process, leading to the production of cytokines [9]. AN seems to be associated with a reduction of the efficacy of innate immunity, especially in terms of decreased chemotaxis and reduced responsiveness of neutrophils [10], as well as with a constant increased level of peripheral inflammation, with higher concentration of interleukin (IL)-6, IL-1β, and tumor necrosis factor (TNF)-α [11]. Considering the possible role of inflammatory processes in AN, changes in IL-21 levels, demonstrated in various types of autoimmune diseases, can also play a significant role. In this context, IL-21 is gaining attention for its powerful modulatory role in many immune cells. IL-21 is a member of the IL-2 family and belongs to the type I, which is produced primarily by lymphocytes B, T, and natural killer T cells, but also by epithelial cells and lamina propria [12]. IL-21 shows widely pleiotropic functions by regulating both adaptive and innate immune responses, as well as exerting key functions in controlling and directing the T and B cell responses, leading to the formation of antibodies. IL-21 is produced by CD4^+^ T cells and allows the lymphocyte differentiation in Th17 to IL-17 for the activation of neutrophils. Furthermore, it plays an important role during the response against the persistent viral infections through CD8^+^ T cell activation. Several human studies have shown that IL-21 can determine Th1 differentiation, improve the expression of transcription factors associated with Th1, and increase the production of IFN-γ. IL-21 can also negatively modulate the maturation and function of DC. In addition to its stimulating effects on T cells, IL-21 is implicated in the maturation and proliferation of B cells, class switching, and the production of antigen-specific antibodies [12].

Thus, a possible role of immune system dysregulation seems to be related to the pathogenesis of AN. Recently, the presence of autoantibodies in AN has been suggested [13]. Actually, it is not easy to determine the action of these antibodies in vivo. The action could be twofold: they could interact with specific ligands expressed by hypothalamic cells, preventing their physiological role, or they could induce non-specific stimulation in target cells, leading to increased secretion of anorexigenic molecules. Further studies are therefore necessary. The production of autoantibodies directed against regulatory peptides and/or hypothalamic neurons could lead to an appetite disorder with reduced food intake. In fact, in subjects with AN, the presence of the autoantibody (Ig)-G is detected against several peptides that regulate appetite [13]. However, the significance of this association often remains difficult to explain. This is because autoantibodies directed against key appetite-regulation peptide hormones or neuropeptides were found in healthy subjects, while psychopathological traits of people with eating disorders are related to the amount and affinities of autoantibodies against anorexigenic and orexigenic neuropeptides. It is possible to assume that the development of AN could be triggered by access to the brain centers of these high-affinity autoantibodies [14]. Further studies are needed to fully understand the involvement of the immune system in AN pathogenesis.

The aim of this study is to describe the inflammatory condition of patients affected by AN compared to controls during different phase of illness, and this is performed at the onset and after three years from the beginning of symptoms. The main hypothesis is that, during the early phase, there are alterations, which promote an inflammatory state able to determine cerebral damage through the production of autoantibodies. Then, the inflammation decreases not only because of the therapy, but also thanks to the establishment of a mechanism of “tolerance”.

## 2. Materials and Methods

### 2.1. Patients’ Enrollment

Between October 2019 and March 2021, 23 patients with AN were recruited according to DSM-5 (Diagnostic and Statistical Manual of Mental Disorders, Fifth Edition) criteria [15]. Among the participants, 17 were affected by AN, restricting subtype, and six were affected by AN, binge-eating/purging subtype. Blood samples were collected in the morning (between 7:30 and 9 a.m.), with last meal at least 6 h before. All participants are female, aged between 18 and 62 (medium age 24 ± 11.2), with a body mass index (BMI) of 16.18 ± 2.04. The control group (CG), paired by age and gender, is composed by 18 females aged between 18 and 51 (medium age 26 ± 9.2) with a BMI of 19.92 ± 0.43, not affected by eating disorder or autoimmune disease. 

Blood serums were stored frozen until the use. Freezing and thawing was avoided.

The Ethical Committee of Istituto di Ricovero e Cura a Carattere Scientifico (IRCCS) Ospedale Policlinico San Martino approved the study (CER 82/13 Emend. 028), and all the participants gave their written informed consent.

The study was carried out according to the Declaration of Helsinki II [16].

### 2.2. Blood Counts

Blood was collected from a forearm vein of participants, as described above, in hemogram tubes containing EDTA. The samples were analysed in the Laboratory of IRCCS (Istituto di Ricovero e Cura a Carattere Scientifico) Ospedale Policlinico San Martino (Genoa). Evaluation of the number of total white blood cells, neutrophils, lymphocytes, monocytes, eosinophils, basophils, and platelets were performed.

### 2.3. Cytokines Evaluation

Specific ELISA kits were used for measuring serum amount of IL-1β, IL-6, TNF-α, and TGF-β, according to the manufacturer’s protocol (Immunotools, Friesoythe, Germany). Each sample was diluted 1:10 and tested in triplicate. Deviation between triplicates was <10% for any reported value. The minimum detectable concentrations were under 23 pg/mL for IL-1β, 8 pg/mL for IL-6, 31 pg/mL for TNF-α, and 31.2 pg/mL for TGF-β. Specific ELISA kits were used for measuring serum IL-21 levels (EMELCA Bioscience, Breda, The Netherlands), according to the manufacturer’s protocol. Each sample was diluted to a ratio of 1:10 and tested in triplicate. Deviation between triplicates was <10% for any reported value. The lowest sensitivity threshold was 0.1 pg/mL.

### 2.4. Anti-Hypothalamus Autoantibody ELISA Protocols

The levels of IgG specific for hypothalamic antigens were measured in the serum of patients with AN and healthy controls by a direct ELISA method built in our laboratory [13]. Briefly, 96-well Maxisorb flat-bottom plates (Nunc) were coated overnight at 4 °C with an available bovine hypothalamic lysate (Science Cell Research, cat#0613) (10 µg/mL in 50 µL/well). After washings, plates were incubated for 2 h with phosphate buffered saline (PBS) −3% bovine serum albumin (BSA) to avoid non-specific interactions. After three washes, 100 µL of 1:100 in PBS−3% BSA diluted serum samples were plated in triplicate and incubated overnight at 4 °C with agitation. After washings, wells were incubated with 100 µL of anti-human IgG HRP-conjugate (Jackson Immune research, as 1:10,000 in PBS−3% BSA buffer) for 45 min at room temperature. Noteworthy, the secondary antibody used is specific to human IgG in order to highlight the totality of autoantibodies to hypothalamic antigens. After incubation, plates were washed, and 100 µL of tetramethylbenzidine (TMB) substrates were added to develop colour for 15 min. Stop Solution (100 µL) was then added, and plates were read at 450 nm for 15–20 min. 

As a part of the assay, a standard curve for human IgG was carried out. It is important to note that this standard curve was not specific for hypothalamic antigens, but exclusively for IgG. A standard curve was obtained by adding 0, 30, 60, 120, 200, 400, 800, and 1200 ng/mL (50 µL) of purified human IgG (Sigma Co. cat# I4506). Standards were performed on the same plate when anti-hypothalamic reactivity levels for serum samples assaying were tested. Upon stopping and washing as described above, anti-human IgG HRP-conjugate (1:10,000 in PBS−3% BSA buffer) was added, followed by TMB substrate addition. Thus, optical density (absorbance) readings of serums from patients with AN and healthy subjects reflect the presence of human IgG against hypothalamic antigens. These readings were converted into IgG concentration in µg/mL, applying a dilution factor of 50 to determine the anti-hypothalamic autoantibodies serum concentrations (50 µL of standards were used, while 1 µL of human serum per sample was loaded per assay). Intra-assay % coefficient of variation (CV) was 5%, and inter-assay % CV was 9.3%. Finally, deviation between triplicates was <10% for any reported value.

### 2.5. Statistical Analysis

Normality distribution of data was verified using the D’Agostino-Pearson normality test [17]. This test computes the skewness and kurtosis to quantify how far the distribution is from Gaussian in terms of asymmetry and shape; then, it calculates how much each of these values differs from the value expected with a Gaussian distribution and computes a single *p* value from the sum of these discrepancies. 

The statistical analysis was carried out using the Mann-Whitney U-test to compare the levels of leukocytes and different cytokines. The Wilcoxon test was performed to analyze differences in cytokine concentration based on the duration of the disease (less or more than three years). Spearman regression analysis has been used to assess the correlation between leukocyte or cytokine levels and BMI in patients with AN. Finally, the possible relationship between the concentration of anti-hypothalamic autoantibodies and the concentration of the different cytokines analysed was evaluated with the Newman-Keuls multiple comparison test. A *p* value of less than 0,05 was considered statistically significant. All analyses were performed using GraphPad Prism 6.0 (GraphPad Software Inc., La Jolla, CA, USA) software.

## 3. Results

### 3.1. Leukocytes

Compared to control group, patients affected by AN show a statistically significant (*p* < 0.001) decreased amount of white blood cells. Results are summarized in Table S1. Primarily altered populations are neutrophils, monocytes, and eosinophils (*p* = 0.001), while lymphocytes and basophils appear reduced, but with a low statistical power (*p* = 0.0046 and *p* = 0.0087, respectively) (Figure S1). Of interest, comparison of the number of these cell population seems to be directly proportional to BMI. This was observed for leukocytes (r = 0.4896, *p* = 0.001), neutrophils (r = 0.4567, *p* = 0.0058), monocytes (r = 0.5893, *p* < 0.0001), and eosinophils (r = 0.5152, *p* = 0.0015), whereas no correlation for lymphocytes (r = −0.0551, *p* = 0.729) and basophils (r = 0.2175, *p* = 0.2025) was observed (Figure S2).

### 3.2. Cytokines 

**Figure 1:**
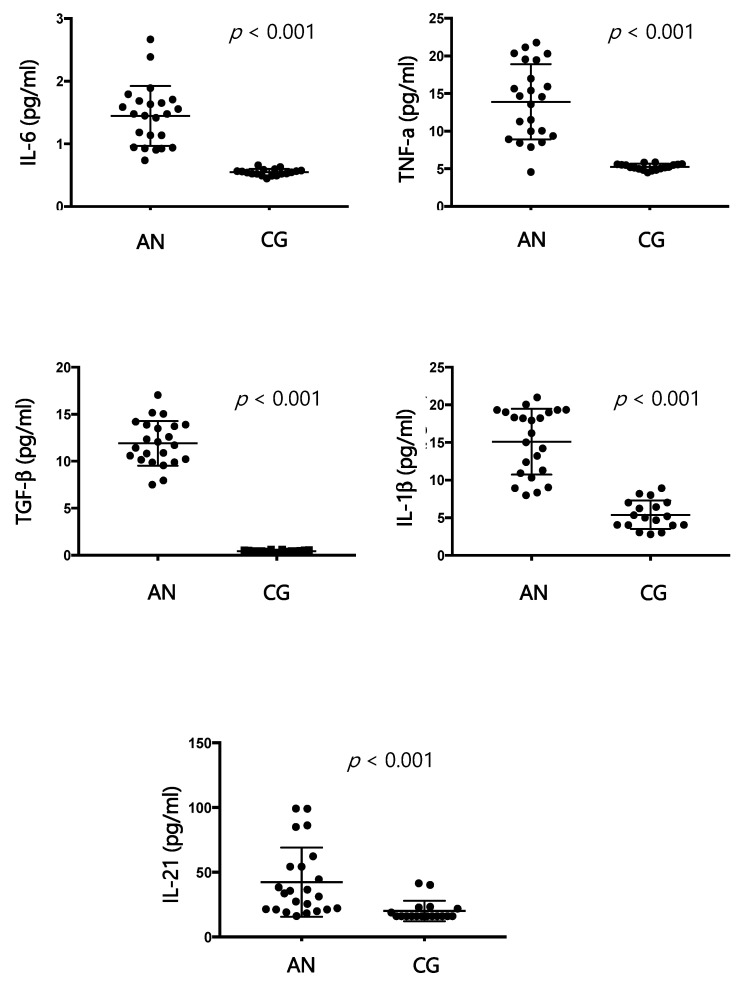
Levels of IL-6, TNF-α, TGF-β, IL-1β, and IL-21 are increased in patients with AN compared to heathy subjects.

Analysis of cytokine concentrations in the patient’s serum showed a marked pro-inflammatory state when compared to control group. In fact, levels of IL-6, TNF-α, TGF-β, IL-1β, and IL-21 are significantly increased in patients with AN compared to heathy subjects. (Figure 1). Results are summarized in Table S2.

### 3.3. Cytokines and BMI 

**Figure 2:**
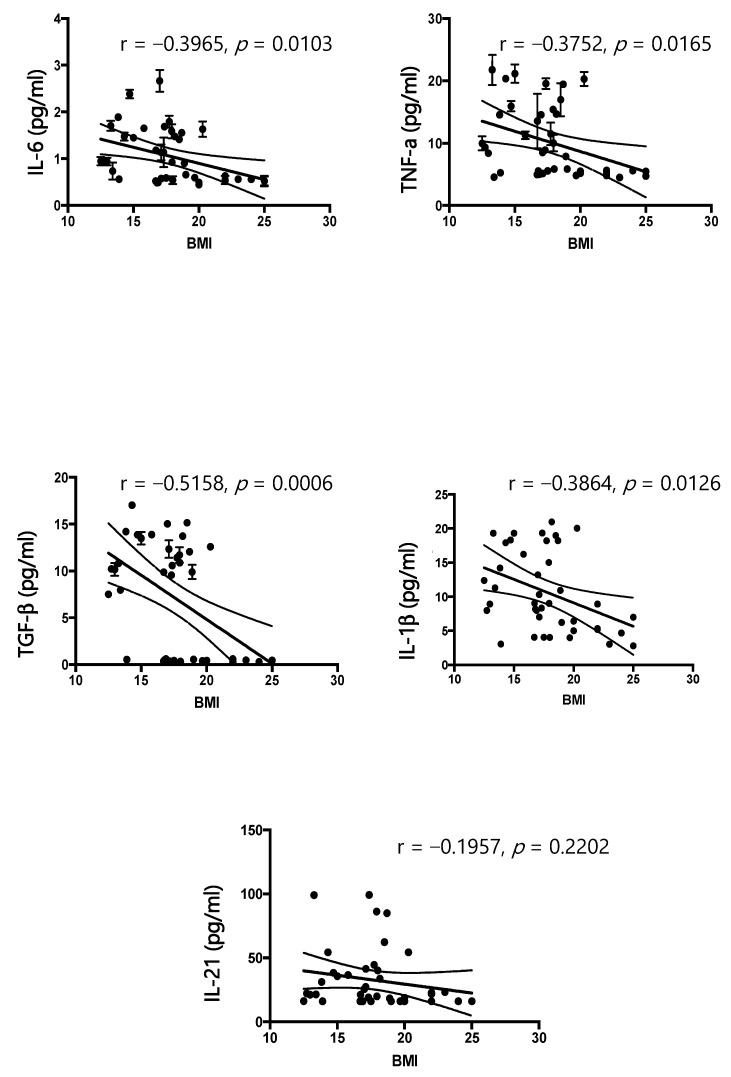
Cytokine levels inversely correlated to BMI of patients with AN.

A correlation between cytokine levels and BMI in patients with AN was investigated. Levels of cytokines are altered in an indirectly proportional way to BMI: the more BMI decreases, the more values of pro-inflammatory cytokines raise (IL-6 r = −0.4745, *p* = 0.0221; TNF-α r = −0.4535, *p* = 0.0297; TGF-β r = −0.4854, *p* = 0.0189; IL-1β r = −0.4176, *p* = 0.0474, and IL-21 r = −0.4963, *p* = 0.0160) (Figure 2).

### 3.4. Cytokines and Duration of Illness

**Figure 3:**
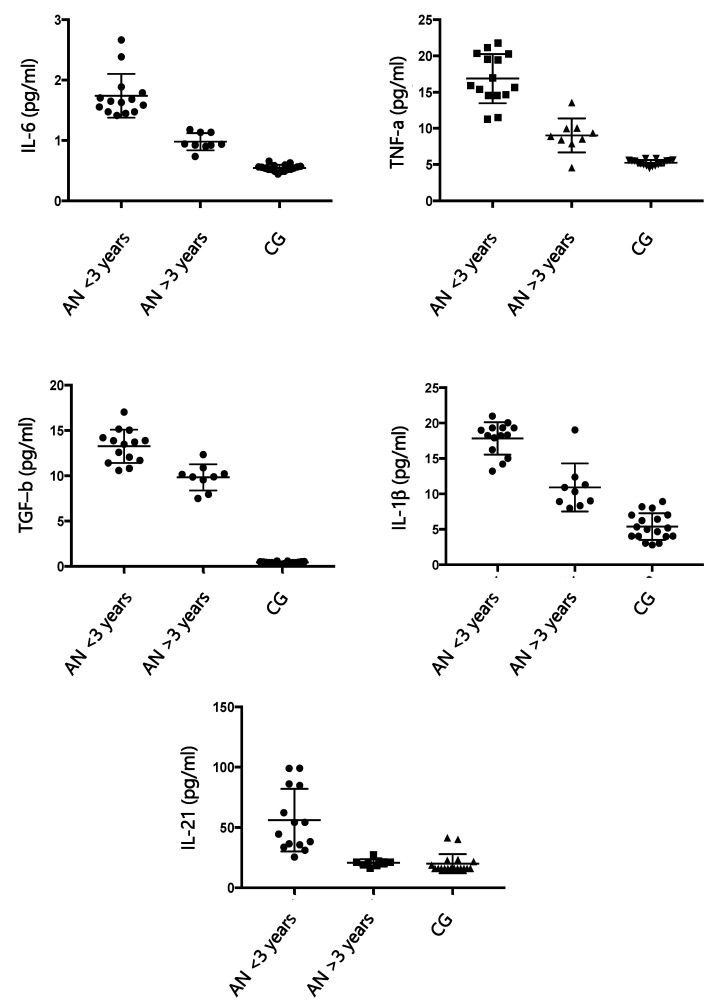
The two populations seem to differ according to cytokines levels and the duration of the disease.

While looking at the graphic distribution of cytokine levels (Figure 1), other markers were evaluated (Figure S3). The possibility of dividing the patients with AN into two different clusters is evident. The patient pool consisted of 14 recently diagnosed patients (under 1.5 years) and nine patients with remote diagnosis (over five years). The observation of the distribution of markers analysed showed that, in the most recent diagnosis group, the values were homogeneous, not as in the group of patients with remote diagnosis (Figure S3). This led us to decide the cut-off of three years. Thus, we hypothesized that the duration of AN can change inflammatory cytokine levels. In fact, levels of cytokines decrease during time, approaching values in healthy controls (Figure 3, and Table S3). In detail, IL-6 AN > three years vs. AN < three years *p* < 0.001; IL-6 AN > three years vs. CG *p* < 0.001; TNF α AN > three years vs. AN < three years *p* = 0.001; TNF-α AN > three years vs. CG *p* = 0.0005; TGF-β AN > three years vs. AN < three years *p* = 0.001, TGF-β AN > three years vs. CG *p* < 0.001; IL-1β AN > three years vs. AN < three years *p* < 0.001; IL-1β AN > three years vs. CG *p* < 0.001; IL-21 AN > three years vs. AN < three years *p* < 0.001; IL-21 AN < three years vs. CG not significant (Figure 3, and Table S3). Finally, the analyses of blood cell numbers in relation to duration of disease does not show a significant statistical correlation (Figure S4). 

### 3.5. Anti-Hypothalamus Autoantibodies

**Figure 4:**
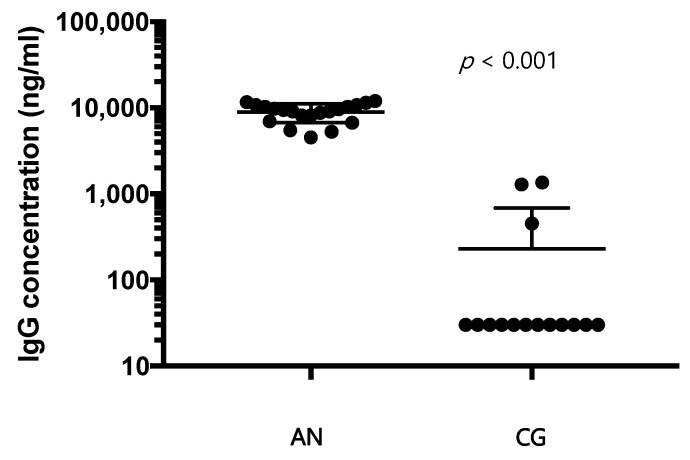
Serum anti-hypothalamus autoantibodies in patients with AN are remarkably increased compared to healthy subjects.

Serum anti-hypothalamus autoantibodies in patients with AN are remarkably increased compared to healthy subjects (mean IgG 8900 ng/mL for patients with AN vs. 44.59 ng/mL for CG, *p* < 0.001) (Figure 4 and Table S4).

### 3.6. Correlation between Anti-Hypothalamus Autoantibodies and Cytokines

**Figure 5:**
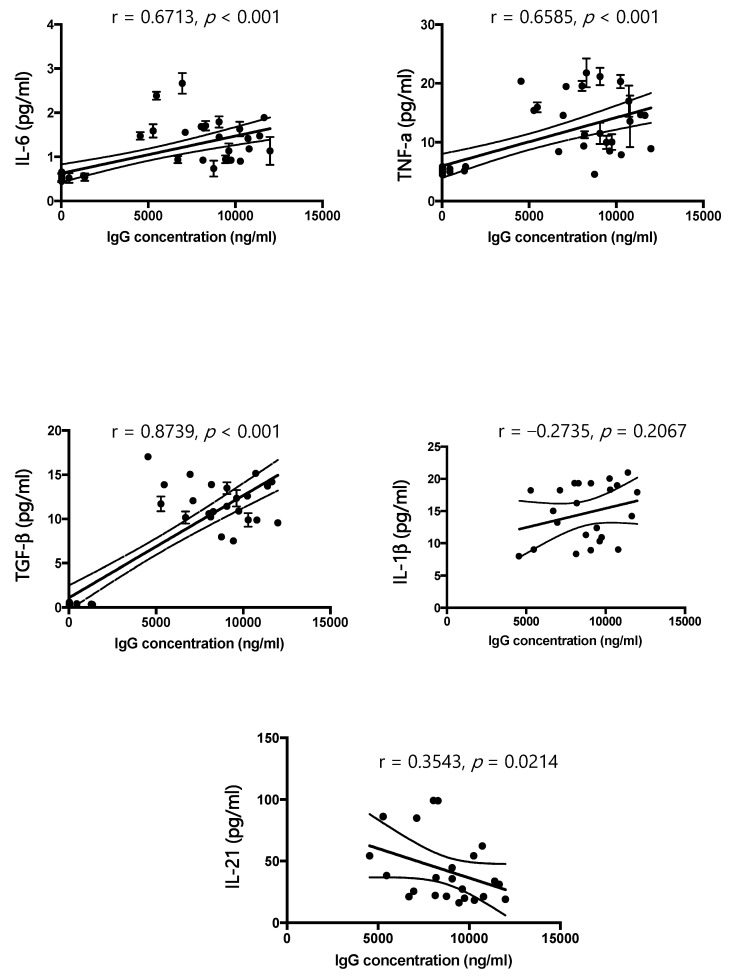
Analysis of IgG and cytokines amounts correlation in sera from patients with AN.

Levels of IL-6, TNF-α, and TGF-β are directly related to concentration of IgG autoantibodies directed against hypothalamic antigens in sera of AN patients; moreover, the amount of autoantibodies reflects the intensity of the inflammatory state (IL-6 r = 0.4570, *p* = 0.0280; TNF-α r = 0.4365, *p* = 0.0373; TGF-β r = 0.3874, *p* = 0.0478). IL-1β shows a potential correlation without any statistical power (r = 0.3945, *p* = 0.0625). On the other side, IL-21 is indirectly related: the less the concentration of this molecule in the blood is, the more levels of anti-hypothalamus IgG increase (r = −0.4207, *p* = 0.0456) (Figure 5). In addition, the concentration of serum autoantibodies to hypothalamic antigens negatively correlate to BMI (r = −0.549, *p* = 0.002), but no correlation to duration of the disease is apparent (r = 0.1836, *p* = 0.402) (Figure S5).

## 4. Discussion

The importance of the immune system in the pathogenesis of a large number of diseases is being increasingly accepted. Although its contribution toward organic disease is easily appreciated, the realization that the immune system is also capable of contributing to the pathogenesis of mental health disorders has only recently become more recognized, as the effects of inflammation on the central nervous system function have been discovered. However, the impact of inflammation toward the development and maintenance of AN has not been fully elucidated. AN, a mental illness characterized by extreme weight loss due to restricted intake resulting from an extreme fear of weight gain, ultimately impacts every organ system and has a very high recidivism rate due to the lack of efficacious treatment options [1,2,3]. If AN is associated with a pro-inflammatory state, as shown in this study, weight restoration, an essential component of treatment, may be much more difficult due to hunger suppression and weight loss effects associated with inflammatory pro-cytokines. On the other hand, in cases where patients can improve their nutritional status, a reduction in the inflammatory state could be also observed.

Plasma of patients with AN reflects the metabolic alterations related to the severe condition of malnutrition. They involve electrolytes, liver enzymes, leukocytic count, haemoglobin, neuropeptide y (NPY), thyroid hormones, follicle-stimulating hormone (FSH) and luteinizing hormone (LH), oestrogens, ghrelin, pancreatic polypeptide, cortisol, and TNF-α [18]. Often, the bone marrow shows sign of atrophy, which rapidly recedes with refeeding [19]. 

Our study highlights a reduction of the leukocytic count in patients with AN compared to healthy controls, especially neutrophils, monocytes, and eosinophils, which is directly related to the decrease in BMI. Thus, it seems that the number of blood cells is approaching normal values with the improvement of the disease. Lymphocytes are the only exception: their number seems to not change over time, at least in the range time we analysed. This characteristic may be related to the lack of change in the concentration of specific autoantibodies for hypothalamic cells with the duration of the disease we observed (Figure S6). Some of these results are supported by the literature [20], and it has also reported how leukocytes, particularly granulocytes, are less reactive in facing inflammation in patients with AN [21]. This could be considered because of the oxidative stress due to the lack of vitamins, which results from undereating [22]. The increase in oxidative stress is, for sure, one of the mechanisms underlying chronic inflammation.

Several authors focused their research on cytokines alterations, especially IL-6, TNF-α, and IL-1β [23]. Cytokines play a role in modulating the hypothalamus and its functions in promoting and regulating appetite [24], as they can influence the catecholaminergic and opioid system [25] and because of their indirect action on the hypothalamic–pituitary–adrenal axis [26]. Moreover, they interact with the production of brain-derived neurotrophic factor (BDNF) and vascular endothelial growth factor (VEGF), as well [27]. It is reasonable to think that all these elements contribute to the genesis of depressive symptoms in those who are affected. 

According to the results of our study, IL-6, IL-1 β, TNF-α, and TGF-β are significantly increased in patients affected by AN compared to healthy controls, and these findings agree with the literature [27,28,29]. Furthermore, our study has demonstrated a positive correlation between cytokine increase and BMI and a progressive reduction of cytokines with the progression of AN. At the beginning of the disease, the pro-inflammatory state could be responsible for the production of autoantibodies directed against the hypothalamus, which can compromise its function [13], causing an excessive release of ghrelin, proopiomelanocortin, and agouti-related protein. In our samples, an excess of IgG directed against the hypothalamus is positively related to the increase in cytokines, and the amount of IgG decreases with the progression of the disease. The duration of the disease appears to be related to a decrease in dysregulation of the immune system. Once again, there could be a two-way relationship between nutritional status and activation of the immune system. In our study, in fact, the improvement of the nutritional condition of patients is accompanied by a rebalancing of the immune system. Reducing the production of specific autoantibodies for hypothalamic cells could also be a sign of improvement in the pathological state. 

AN and autoimmune diseases appear to be linked by a two-way relationship based on common immunopathological mechanisms [30]. There is increasing evidence that people suffering from autoimmune diseases may develop AN and vice versa. Thus, the presence of an inflammatory state, alterations in the immune response, and the production of autoantibodies would be the common pathogenetic substrates [30]. However, to date, there are no systematic studies that can accurately define the common immunopathogenetic mechanisms and links between autoimmunity and eating disorders and establish the mutual risk of transition from one disease to another. If these results will be confirmed, AN should be reconceptualized as a psycho-neuro-endocrine-immune disorder. It is probably not easy to clarify the role of the immune component. However, focusing on both neuro-psychiatric and immune components may be the key to improving outcomes.

The results of this study show a relationship between AN and inflammation, which is probably bidirectional. In fact, it has been demonstrated that immune and inflammatory abnormalities can increase the risk of developing eating disorders; at the same time, those suffering from eating disorders have an increased risk of developing an autoimmune disease (such as Hashimoto’s thyroiditis or Crohn’s disease) [28].

The close correlation between pro-inflammatory cytokines, the nervous system, and loss of appetite seems to be directly related to an increased risk of infections, cardiovascular conditions, and depressive symptoms [27,31].

Recent studies, which show the presence of immune dysregulation in anorexia as well as in psychiatric disorders [13,32], lead us to speculate on the role of autoimmunity in these pathological conditions. In particular, it has been pointed out that eating disorders, similar to several other psychiatric conditions, are associated with the presence of autoantibodies [13]. It is known that a relatively large number of brain antigens are implicated in autoimmune diseases, and this has suggested a strong evolutionary link between the immune system and the central nervous system that has bidirectionally shaped the cooperation between these two systems [30]. Recently, we have highlighted an immune-reactivity toward primate hypothalamic neurons in serum from subjects with AN, suggesting an underlying autoimmunity role [13]. These autoantibodies may be able to determine an appetite disorder with the result of reduced food intake. 

An innovative finding of this study is the observation that the concentration of IL-21 is increased in the blood of patients with AN. IL-21 is an autocrine cytokine produced by follicular T-cells and T-helper cells, which plays a wide action, depending on environmental signals and can act by modulating both humoral and cellular immune responses [33,34,35]. In fact, it promotes the proliferation, the development, and the activation of T-cell subsets (both helper and cytotoxic), inducing the generation and differentiation of B-cells into plasma cells and enhancing the production of immunoglobulins [33,34,35]. According to the hypothesis of a bidirectionality between autoimmune diseases and AN, the role of IL-21 seems to be important also in the pathogenesis of AN. Previous results have shown an increase in IL-21 in the serum of celiac patients and the correlation of the amount of this cytokine with damage to the duodenal mucosa and production of autoantibodies [33]. In addition, overexpression of IL-21 neutralises the suppressive capability of Treg cells with the development of type 1 diabetes [36]. In the AN context, given the variety of effects that IL-21 has on the immune system, IL-21 could act as a stimulant of the immune response determining the initiation and progression of inflammatory reactions involved in several autoimmune diseases [33].

The possible role of immune system disruptors in autoimmune diseases and eating disorders appears to be increasingly well defined. A high prevalence of autoimmune diseases is reported among patients with eating disorders [13]. So, there may be shared immunological pathways that connect autoimmune and food diseases. Autoimmunity could trigger and/or suppress the eating disorder, at least in a group of patients (which we are currently unable to define molecularly). Cases of AN associated with juvenile systemic lupus erythematosus [37], Hashimoto’s thyroiditis [38], celiac disease [39], and inflammatory bowel disease [40] have been recently demonstrated.

Limitations of our study include, principally, the small sample, especially regarding men that were not enrolled, which does not allow for generalization of the findings. Of note, eating disorders in men have been almost ignored for many years and until recently have been typically seen as a female problem. Today, eating disorders (anorexia, bulimia, and especially binge-eating) are also increasing in the male population [41,42]. In addition, a possible limitation of this study is its cross-sectional design. Thus, possible effects related to weight gain and treatments could not clearly differentiate the analysed immunological parameters. Therefore, we aim to expand the study to better understand if the measured immunological parameters are linked to etiological factors or if they are not rather a corresponding immunological reaction to low weight and malnutrition.

Considering the enrollment of patients at different time points, it was not possible to investigate the different phase of the disease through specific validated scales. Since it is quite rare to obtain laboratory data during the premorbid stage, it could be useful to study the health history to identify immune alterations in the first-degree relatives of the affected subjects to evaluate whether the manifestations of the patients are part of a cluster shared among the parental structures.

## 5. Conclusions

This study shows that the increased pro-inflammatory phenotype in patients with AN correlates with the concentration of autoantibody specific for hypothalamic antigens. Of interest, the inflammatory state seems to be reduced with duration of AN. In addition, IL-21 could act as a stimulant of the immune response, thus increasing the possible autoimmune effect. Known its regulatory role, IL-21 could be used as a therapeutic target: the development of compounds neutralizing IL-21 (such as blocking antibodies or recombinant proteins) constitutes an exciting therapeutic arm for autoimmune diseases. 

The more studies are carried out, the greater the role of inflammation control as an overall therapeutic benefit seems to be, regardless of whether it is secondary to early trauma, a more acute stress response, microbiome alterations, a genetic diathesis, or a combination of these and other factors. In fact, the correlation between pro-inflammatory cytokines, nervous system, and loss of appetite seems to be directly related to an increased risk of infections, cardiovascular conditions, and depressive symptoms. 

Of interest, the concentration of autoantibodies directed to hypothalamic antigens does not appear to decrease significantly with the duration of the disease. We could hypothesize that, since the putative hypothalamic autoantigens are still present, a basal stimulation to their production is functionally active.

Further studies are needed to fully understand the role of immune system in AN pathogenesis and to identify parameters that could help to describe the entire course of disease.

## Data Availability

Data that support the findings of this study and materials are available from the corresponding author upon request.

## Supplementary Materials

The following supporting information can be downloaded at: https://www.mdpi.com/article/10.3390/nu15092199/s1, Figure S1: Patients affected by AN show a decreased amount of white blood cells; Figure S2: Analyses of correlation among white cell sub-population and BMI; Figure S3: The analyses of different markers (blood cells upper panels, and cytokines and BMI lower panels) in relation to duration of disease showed an apparent cluster of patients with AN in two subpopulations. We choose arbitrary a cut-off of three years; Figure S4: The analyses of blood cells concentration do not seem to be statistically related to the duration of disease (*p* > 0.05 for all the markers showed); Figure S5: The amount of autoantibodies to hypothalamic antigens decreases with the increase of BMI. On the contrary, no relation with the duration of disease seems to exist. Figure S6: The number of lymphocytes is increased in patients with AN and does not change significantly during time. The relationship to the amount of IgG autoantibodies directed to hypothalamic antigens is apparent, even if not statistically significant (*p* = 0.09); Table S1: Analyses of total leukocytes and different population in AN patients and control group (CG); Table S2: Serum cytokines amount in patients with AN and in healthy donors (CG); Table S3: Serum cytokine amounts and BMI changing in AN during time and comparison with CG; Table S4: Serum anti-hypothalamus autoantibodies in patients with AN are remarkably increased compared to control group (CG).
