# Supplementary material for: Dysfunction of Inflammatory Pathways and Their Relationship with Anti-Hypothalamic Autoantibodies in Patients with Anorexia Nervosa
Source: Nutrients. 2023 May 5;15(9):2199. doi: 10.3390/nu15092199 (PMC10180712; doi:10.3390/nu15092199)
Supplement: Supplementary file 1 [file nutrients-15-02199-s001.zip › nutrients-2315913-supplementary.pdf]

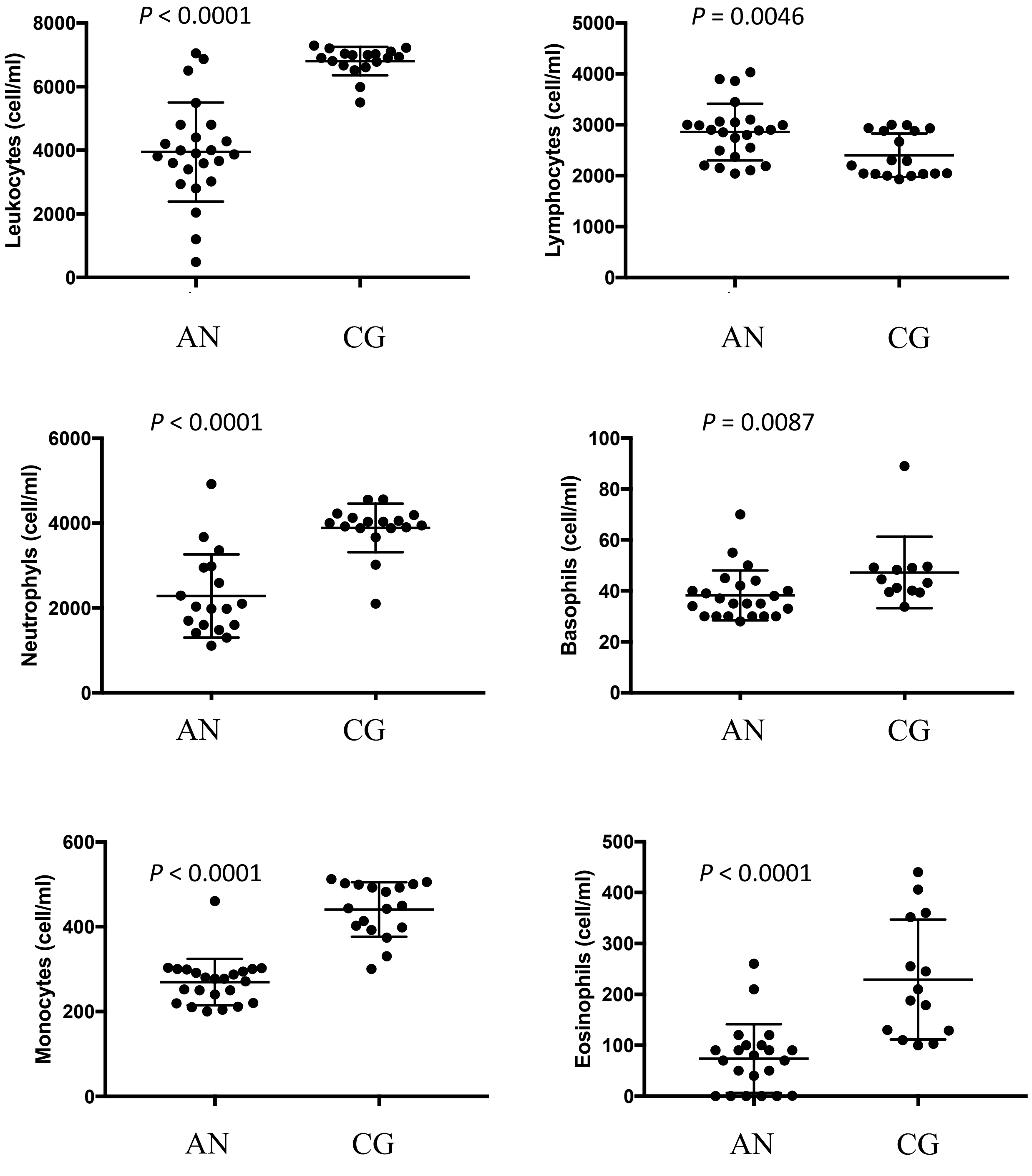


**Figure S1.** Patients affected by AN showed a decreased amount of white blood cells, except lymphocytes that are increased.

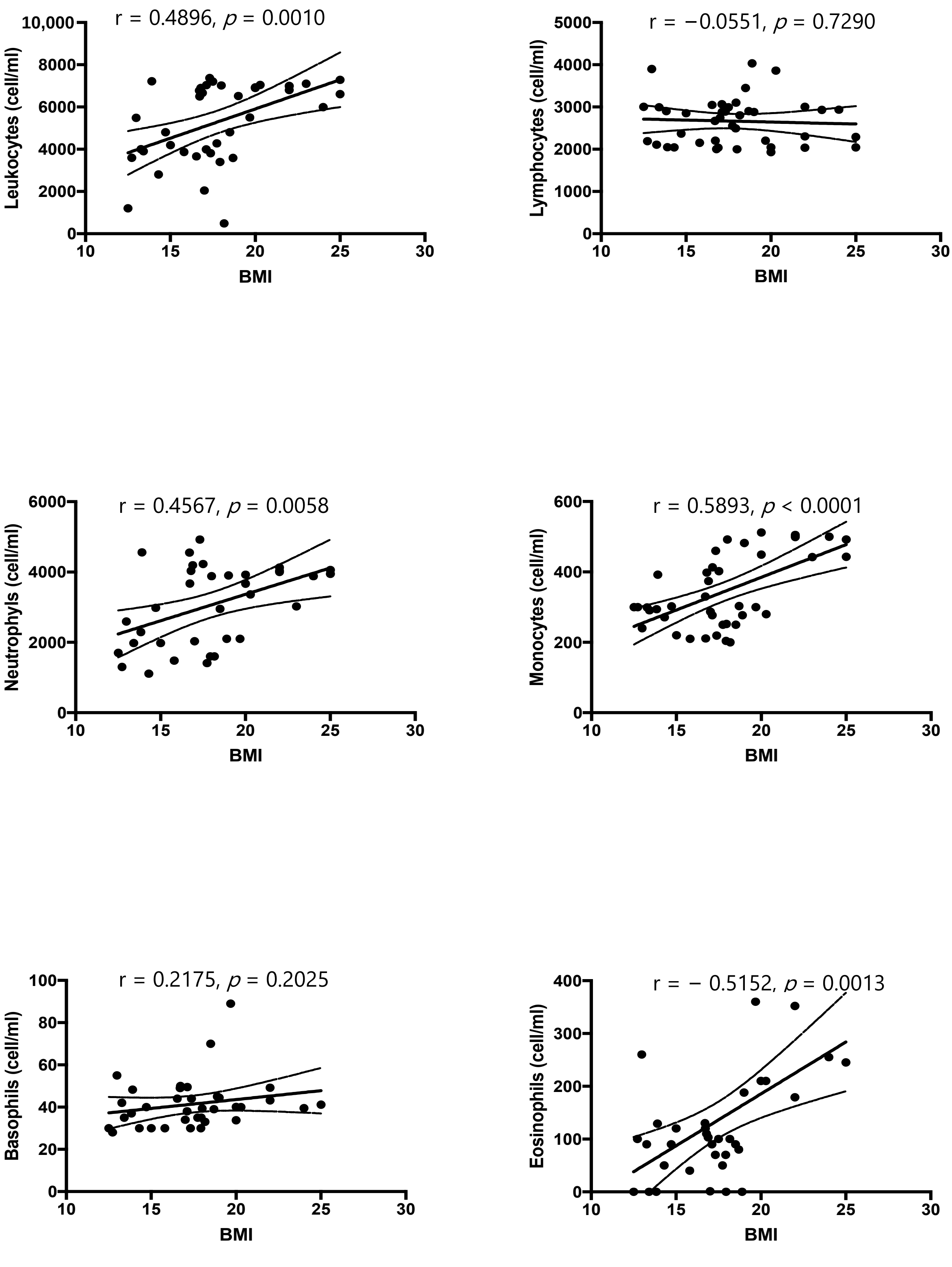


**Figure S2.** Analyses of correlation among white cell sub-population and BMI in both AN and control group.

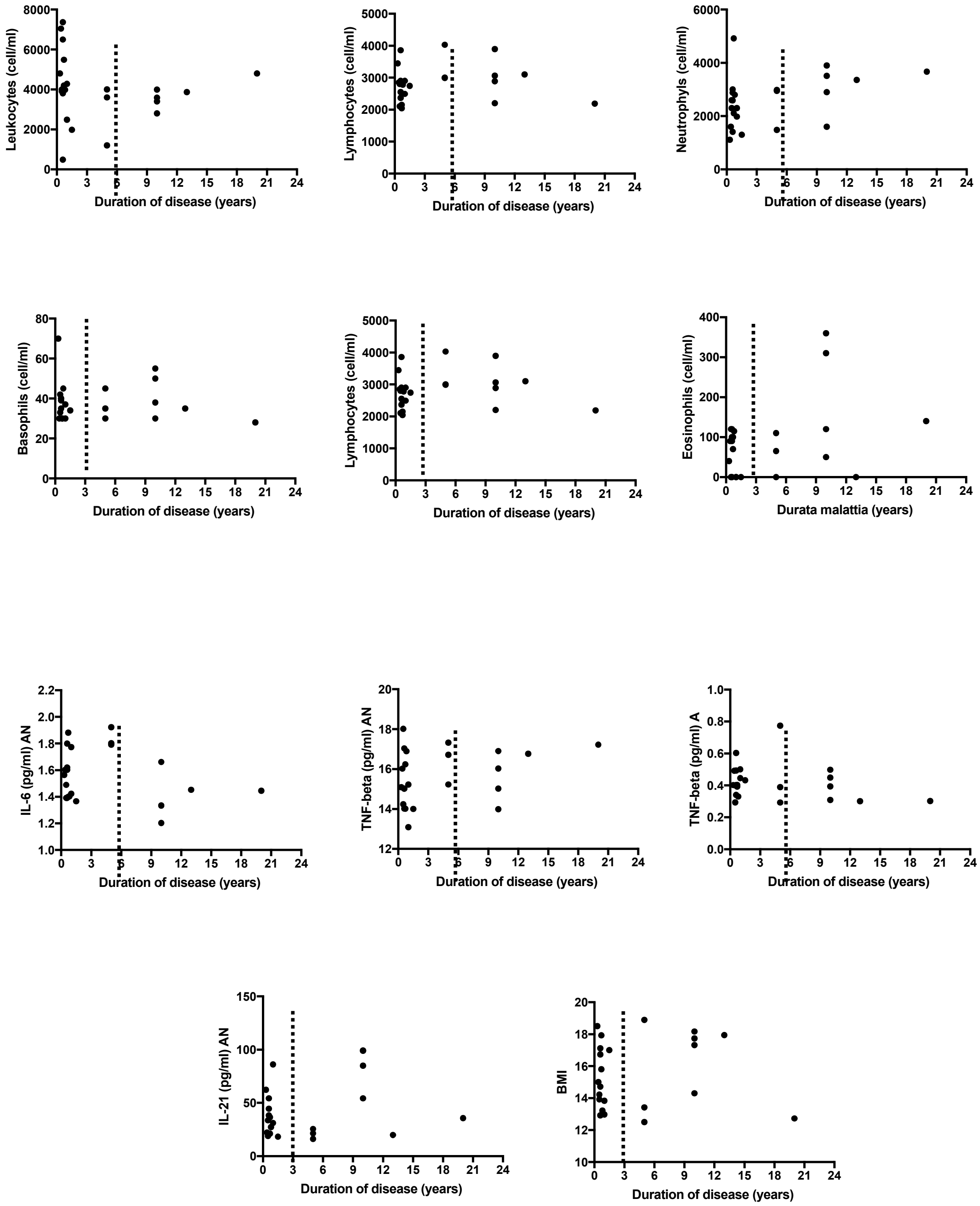


**Figure S3.** The analyses of different markers (blood cells upper panels, and cytokines and BMI lower panels) in relation to duration of disease showed an apparent cluster of AN patients in two subpopulations. We choose arbitrary a cut-off of 3 years.

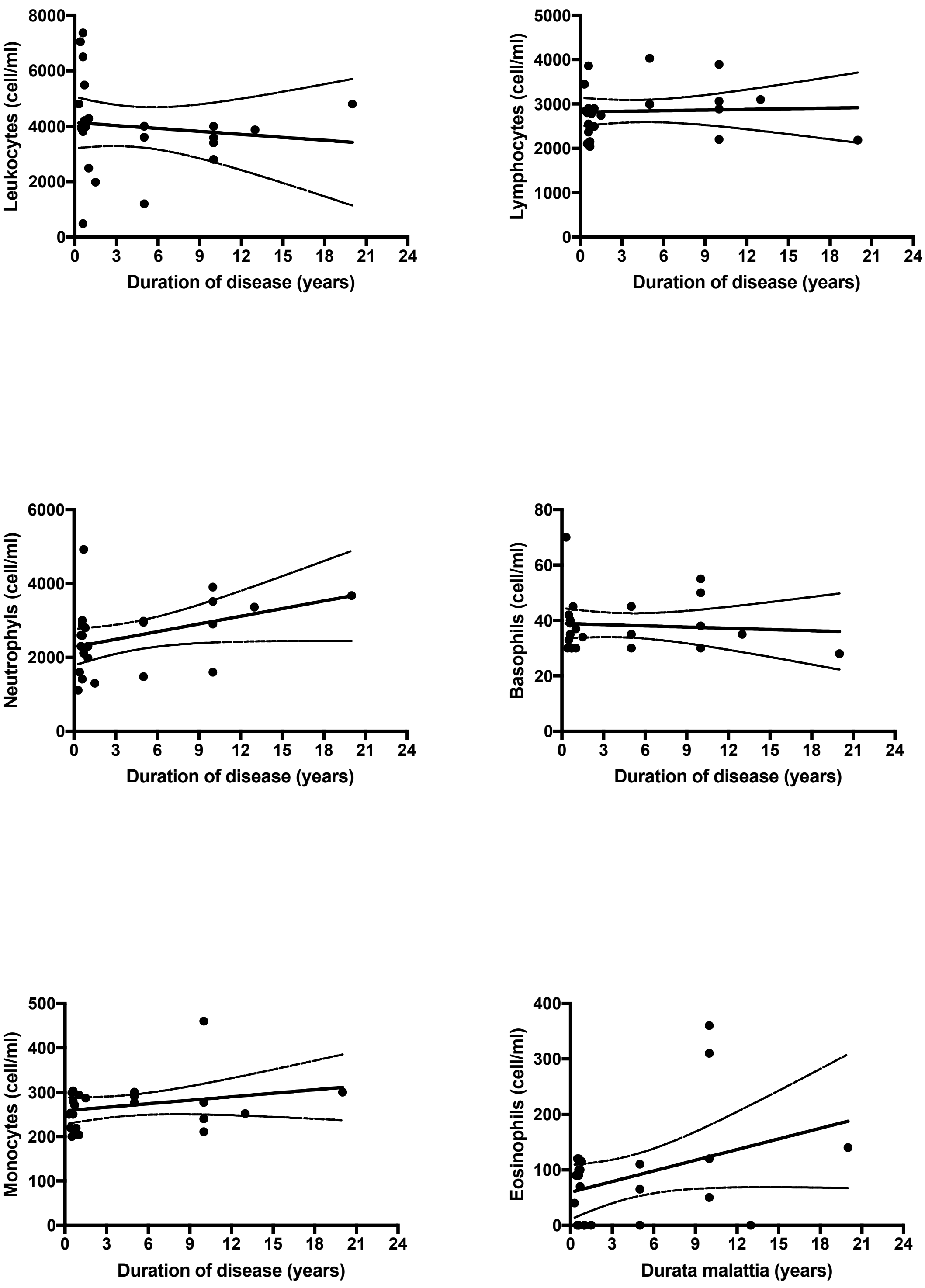


**Figure S4.** The analyses of blood cells concentration do not seems to be statistically related to the duration of disease ($p>0.05$ for all the markers showed).

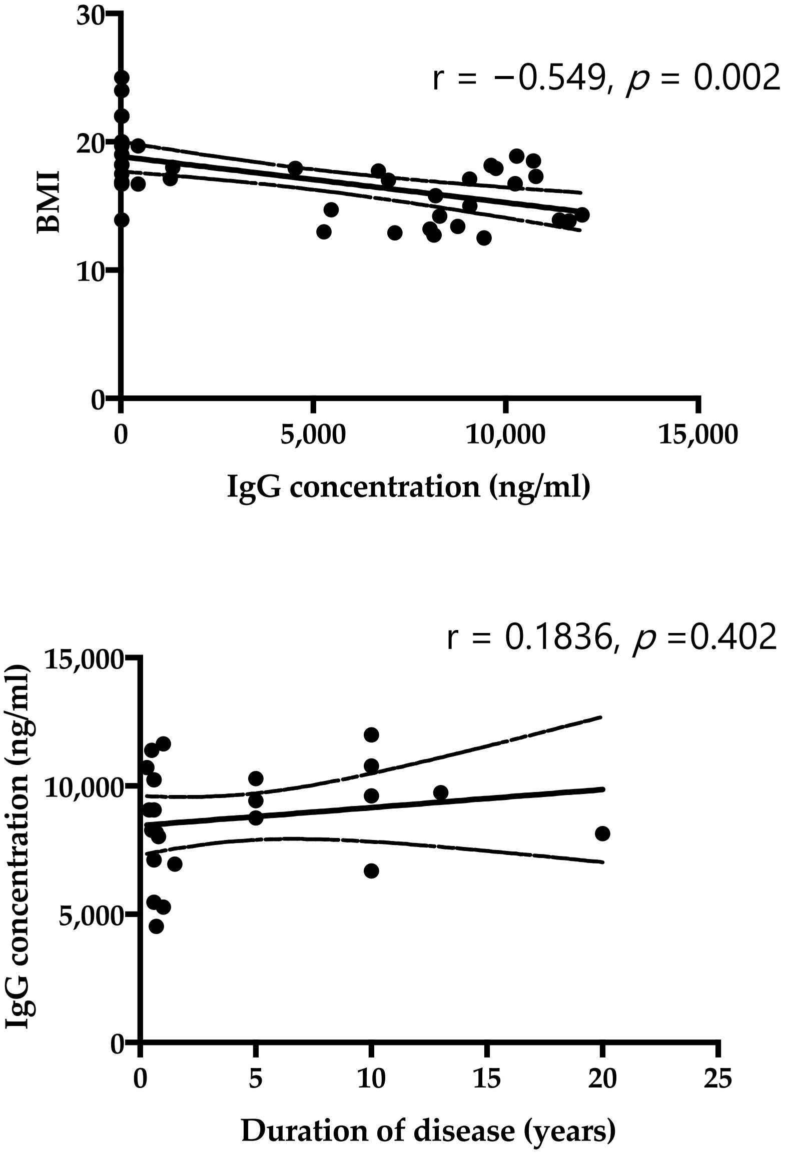


**Figure S5.** The amount of autoantibodies to hypothalamic antigens decreases with the increase of BMI. On the contrary, no relation with the duration of disease seems to exist.

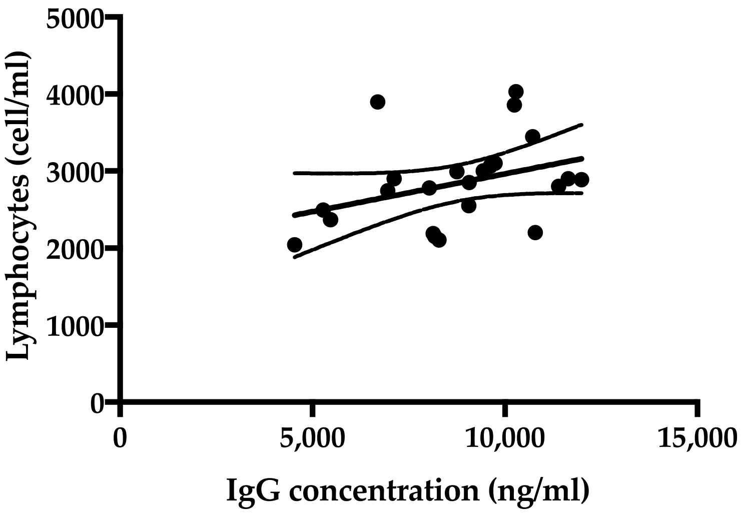


**Figure S6.** The number of lymphocytes is increased in AN patients and does not change significantly during time. The relationship to the amount of IgG autoantibodies directed to hypothalamic antigens is apparent, even if not statistically significant (*p* = 0.09).

**Table S1.** Analyses of total leukocytes and different population in AN patients and healthy controls (CG).

| | AN Mean ±Standard dev. | | CG Mean ±Standard dev. | | p value* |
|---|---|---|---|---|---|
| Leukocytes (cell/ml) | 3947 | ±1559 | 6805 | ±445 | <0.0001 |
| Lymphocytes (cell/ml) | 2860 | ±556 | 2401 | ±425 | 0.0046 |
| Neutrophils (cell/ml) | 2281 | ±981 | 3889 | ±572 | <0.0001 |
| Monocytes (cell/ml) | 269 | ±55 | 440 | ±38 | <0.0001 |
| Basophils (cell/ml) | 38 | ±9 | 47 | ±14 | 0.0087 |
| Eosinophils (cell/ml) | 74 | ±67 | 229 | ±118 | <0.0001 |

* indicates AN patients vs CG differences (p<0.05 significative).

**Table S2.** Serum cytokines amount in AN patients and in healthy donors (CG).

| | IL-6 (pg/ml) | | TNF-α (pg/ml) | | TGF-β (pg/ml) | | IL-1β (pg/ml) | | IL-21 (pg/ml) | |
|---|---|---|---|---|---|---|---|---|---|---|
| | AN | CG | AN | CG | AN | CG | AN | CG | AN | CG |
| Minimum | 0.73 | 0.45 | 4.57 | 4.51 | 7.51 | 0.26 | 7.99 | 2.80 | 16.20 | 16 |
| Maximum | 2.67 | 0.66 | 21.78 | 5.87 | 17.04 | 0.60 | 20.99 | 8.92 | 99.20 | 41.4 |
| Median | 1.48 | 0.54 | 14.56 | 5.23 | 11.72 | 0.43 | 16.23 | 5.10 | 33.70 | 16 |
| | | | | | | | | | | |
| Mean | 1.44 | 0.55 | 13.91 | 5.27 | 11.92 | 0.44 | 15.12 | 5.39 | 42.30 | 20.02 |
| Std. Deviation | 0.48 | 0.05 | 5.00 | 0.39 | 2.38 | 0.10 | 4.37 | 1.88 | 26.73 | 7.976 |

**Table S3.** Serum cytokine amounts and BMI changing in AN during time and comparison with CG.

| | IL-6 (pg/ml) | | IL-6 (pg/ml) | TNF-α (pg/ml) | | TNF-α (pg/ml) | TGF-β (pg/ml) | | TGF-β (pg/ml) |
|---|---|---|---|---|---|---|---|---|---|
| | AN <3year | AN >3year | CG | AN <3year | AN >3year | CG | AN <3year | AN >3year | CG |
| Minimum | 1.42 | 0.74 | 0.45 | 11.28 | 4.57 | 4.51 | 10.59 | 7.51 | 0.26 |
| Maximum | 2.67 | 1.18 | 0.66 | 21.78 | 13.57 | 5.87 | 17.04 | 12.34 | 0.60 |
| Median | 1.64 | 0.94 | 0.54 | 15.93 | 8.92 | 5.23 | 13.61 | 9.89 | 0.43 |
| Mean | 1.74 | 0.98 | 0.55 | 16.88 | 9.03 | 5.27 | 13.26 | 9.83 | 0.44 |
| Std. Deviation | 0.36 | 0.14 | 0.05 | 3.39 | 2.36 | 0.39 | 1.834 | 1.44 | 0.10 |

| | IL-1β (pg/ml) | | IL-1β (pg/ml) | IL-21 (pg/ml) | | IL-21 (pg/ml) | BMI | | BMI |
|---|---|---|---|---|---|---|---|---|---|
| | AN <3year | AN >3year | CG | AN <3year | AN >3year | CG | AN <3year | AN <3year | CG |
| Minimum | 13.22 | 7.993 | 2.8 | 25.6 | 16.2 | 16.00 | 12 | 12.5 | 13.9 |
| Maximum | 20.99 | 19.03 | 8.92 | 99.2 | 27.33 | 41.4 | 18.5 | 18.9 | 25 |
| Median | 18.28 | 10.32 | 5.103 | 49.45 | 21.2 | 16.00 | 15 | 17.3 | 19 |
| Mean | 17.82 | 10.92 | 5.39 | 56.14 | 20.76 | 20.02 | 15.19 | 15.89 | 19.24 |
| Std. Deviation | 2.29 | 3.38 | 1.88 | 25.99 | 3.09 | 7.98 | 2.09 | 2.6 | 3.02 |

**Table S4.** Serum anti-hypothalamus autoantibodies in patients with AN are remarkably increased compared to healthy subjects (CG).

| | IgG anti-hypothalamic antigens concentration (ng/ml) | |
|---|---|---|
| | AN | CG |
| Minimum | 4,534 | 30 |
| Maximum | 11,988 | 135 |
| Median | 9,255 | 30 |
| Mean | 8,900 | 44.59 |
| Std. Deviation | 2,167 | 35.68 |
